# Barriers, Facilitators, and Strategies for Sustaining the Hospital-Wide “One Bed” Model in China: A Single-Centre Descriptive Qualitative Study of Healthcare Professionals’ Perspectives

**DOI:** 10.3390/healthcare14162595

**Published:** 2026-08-18

**Authors:** Hongfan Yin, Jingjing Fu, Xiaomei Chen, Min Chen, Ting Yin, Xuting Zhang, Huiqin Xi, Liuyun Yu

**Affiliations:** 1 Department of Nursing, Renji Hospital, Shanghai Jiao Tong University School of Medicine, Shanghai 200127, China; yinhongfan@renji.com (H.Y.); renji_fjj@126.com (J.F.); chenxiaomei@renji.com (X.C.); renji_cm@126.com (M.C.); yinting@renji.com (T.Y.); zhangxuting@renji.com (X.Z.); 2 School of Nursing, Shanghai Jiao Tong University, No. 227, South Chongqing Road, Shanghai 200025, China

**Keywords:** centralized hospital services, hospital bed capacity, delivery of health care, integrated, qualitative research, workload

## Abstract

**Highlights:**

**What are the main findings?**

- Hospital-wide bed sharing was perceived to improve access to beds, but it also redistributed workload and hidden coordination work to receiving wards.
- Bed integration outpaced care integration: patients moved faster than medical response, nursing readiness, functional support, and governance arrangements could adapt.

**What are the implications of the main findings?**

- Bed-sharing models should be evaluated beyond bed occupancy, turnover, and waiting time, with attention to nursing workload, clinical suitability, and coordination work.
- Sustainable implementation requires bounded flexibility, including specialty fit, timely physician response, cross-specialty nursing support, functional systems, and shared accountability.

**Abstract:**

**Background**: Hospital-wide centralized bed allocation, known in China as the “one-bed-for-the-whole-hospital” model, aims to improve inpatient access by pooling beds across specialties. Existing studies have mainly examined operational outcomes, process risks, or staff competence. Less is known about how bed redistribution interacts with clinical responsibility, professional roles, functional support, and shared governance. **Objectives**: This study explored healthcare professionals’ perspectives on the barriers, facilitators, and strategies for sustaining the hospital-wide “One Bed” model. It also examined how bed integration and care integration aligned or diverged across the dimensions of the Rainbow Model of Integrated Care. **Methods**: A single-center descriptive qualitative study was conducted in a Grade A tertiary hospital in Shanghai, China, where the model had operated across 11 pilot wards for approximately 48 months. Between December 2024 and March 2025, 25 healthcare professionals, including 14 clinical nurses, eight head nurses, and three physicians, completed face-to-face semi-structured interviews. Data were analyzed using inductive qualitative content analysis. After themes and subthemes were developed from participants’ accounts, the Rainbow Model of Integrated Care was used as an interpretive framework to map the findings across clinical, professional, organizational, system, functional, and normative integration. **Results**: Five themes were generated. Participants perceived centralized bed allocation as shortening waiting time and improving bed use, but also as intensifying ward workload and making single efficiency indicators insufficient. Patients could move to available wards before medical response, responsibility, and physician visibility were fully aligned. Cross-specialty case mixes exceeded what nurses could manage through temporary learning alone. Information, logistics, space, equipment, and supplies did not always move with patients, leaving nurses to maintain workflow through manual and often invisible coordination. Sustained bed sharing also depended on clearer boundaries for patient selection, specialty fit, severity, nursing workload, leadership authority, resources, and incentives. Together, these themes showed that bed resources were integrated faster than care processes, professional capability, functional support, and shared governance. **Conclusions**: Hospital-wide centralized bed allocation should be understood as an uneven process of integration rather than only as a bed-management strategy. These findings primarily reflect the perceptions and experiences of nurses and nursing managers, supplemented by limited physician input. The distinctive finding of this study is that beds may be pooled, and patients may move rapidly, while medical response, nursing competence, functional support, workload recognition, and governance arrangements do not always move at the same pace. Safe and sustainable implementation therefore requires bounded flexibility: bed allocation should be guided not only by bed vacancy but also by clinical suitability, specialty fit, nursing workload, timely medical response, functional systems that move with patients, and shared accountability.

## 1. Introduction

Timely access to inpatient beds is essential for hospital care. However, admission demand varies across time and specialties, whereas beds are usually allocated within fixed departmental boundaries. When demand and bed allocation do not align, some specialties become congested, while beds remain available elsewhere. Patients may consequently experience delayed admission. Hospital access therefore depends not only on total bed capacity but also on whether beds can be redistributed promptly. Patient flow is also shaped by capacity planning, decision-making, teamwork, and information exchange. It should therefore be understood from a hospital-wide rather than department-specific perspective [1]. To improve bed use, Chinese policy now encourages centralized management and hospital-wide allocation of available beds [2].

International policy places hospital efficiency within the broader goals of integrated, people-centered, and high-quality care. The World Health Organization (WHO) Framework on Integrated, People-Centred Health Services calls for services that are coordinated across care settings and are comprehensive, safe, effective, timely, efficient, and acceptable. It also emphasizes that health workers should be appropriately skilled, motivated, and supported within their working environment [3]. At the regional level, the WHO Regional Action Framework on Improving Hospital Planning and Management in the Western Pacific identifies accountability, efficiency, quality, equity, sustainability, and resilience as interrelated dimensions of hospital performance. It recommends strengthening patient flow, logistics, human-resource management, clinical governance, and patient safety rather than pursuing operational efficiency in isolation [4]. Although these WHO frameworks do not prescribe a specific hospital-wide bed-sharing model, they provide relevant principles against which such organizational reforms can be considered [3,4].

Against this background, Chinese hospitals have introduced the hospital-wide bed-sharing model, commonly known as the “one-bed-for-the-whole-hospital” model. This model removes the fixed link between a bed and a specialty. When no bed is available in the original specialty, a patient may be admitted to another unit, termed the host ward. The original specialty usually retains responsibility for diagnosis and treatment, whereas the host ward provides daily nursing care. Single-center evidence suggests that the model may shorten admission waits, improve bed use, and enhance the service experience [5]. However, the patient’s location, medical team, and nursing team no longer fully overlap. Bed redistribution, therefore, does not ensure that clinical responsibility, care processes, and interdepartmental collaboration are aligned. Qualitative evidence has identified changes in physicians’ work, a greater nursing burden, communication barriers, clinical risks, and differences in patient experience [6]. In broader outlying-patient settings, safety concerns also involve unclear accountability, reduced access to specialists, inconsistent nursing expertise, and disrupted information transfer [7]. Thus, improved bed use may be accompanied by greater difficulty coordinating care.

These considerations are particularly relevant to nursing because cross-ward placement may alter patient case mix, staffing demands, interprofessional relationships, and nurses’ access to managerial and specialist support. In the WHO Global Strategic Directions for Nursing and Midwifery 2021–2025, education, jobs, leadership, and service delivery are identified as interrelated policy priorities for strengthening the nursing workforce [8]. The WHO Global Patient Safety Action Plan 2021–2030 further treats avoidable harm as a system-level concern that requires coordinated action through policy, organizational processes, workforce capability, and point-of-care practice [9]. Multinational evidence also indicates that nurse staffing and features of the nursing practice environment—including managerial support, nurse participation in decision-making, nurse–physician relationships, and organizational priorities for care quality—are associated with patient satisfaction, perceived quality and safety of care, and nursing workforce outcomes [10]. Hospital-wide bed sharing should therefore be considered not only in relation to bed utilization, admission waiting time, and workload, but also in relation to nursing care quality, the nursing practice environment, and patient safety. However, little is known about how healthcare professionals perceive these dimensions to interact when patients and bed capacity are redistributed across specialty boundaries.

Existing responses have mainly addressed process risks and staff competence. Failure Mode and Effects Analysis has been used to identify vulnerabilities in cross-departmental admission. Targeted training may also strengthen nurses’ cross-specialty knowledge and competence [11,12]. These measures address specific risks, but their scope remains local. The central questions extend beyond whether nurses possess adequate specialty knowledge. They also concern who reassesses the patient, how treatment decisions are communicated, how responsibilities are divided, and how cross-departmental work is recognized. If bed-allocation rules change while accountability, information systems, staffing, and performance arrangements remain department-based, beds may be shared while care remains fragmented. The model should therefore be examined as a hospital-level redesign of management and care delivery, rather than as a collection of individual competence problems [1,6,7].

The Rainbow Model of Integrated Care (RMIC) provides a suitable framework for this analysis. It distinguishes clinical integration at the micro level, professional and organizational integration at the meso level, and system integration at the macro level [13,14]. Functional and normative integration connect these levels through information, financial, and managerial support, and shared values [13,14]. This structure closely reflects the challenges of hospital-wide bed sharing. Centralized bed allocation may establish organizational integration before clinical coordination, professional accountability, and supporting mechanisms are fully developed. RMIC has been applied to integrated care for older adults with multimorbidity in China and to psycho-social integration in specialty care [15,16]. These applications support its use for organizing experiences across multiple actors and levels. However, available evidence on hospital-wide bed sharing has mainly addressed operational outcomes, staff experiences, process risks, and training. Less is known about how integration dimensions interact or why resource integration may precede care integration. Clarifying this process may explain why improved bed efficiency can coexist with fragmented care.

Accordingly, this qualitative study explored healthcare professionals’ perspectives on the barriers, facilitators, and strategies for sustaining the hospital-wide “One Bed” model. Participants included physicians, nurse managers, and clinical nurses. RMIC was used as an interpretive framework to examine perceived benefits, problems, and enabling conditions across its six integration dimensions. The study further examined how these dimensions interacted and where coordination remained incomplete. Rather than evaluating effectiveness alone, the study sought to explain how the model operated and identify conditions needed for safe, continuous, and person-centered care.

## 2. Materials and Methods

### 2.1. Study Design

We conducted a single-center descriptive qualitative study to explore barriers, facilitators, and strategies for sustaining the hospital-wide “One Bed” model from the perspectives of nurses, physicians, and head nurses. The RMIC informed the development of the interview guide and was subsequently used as an interpretive framework for understanding the inductively developed findings. The RMIC encompasses six domains across the micro-, meso-, and macro-levels: clinical, professional, organizational, system, functional, and normative integration. Data were collected between December 2024 and March 2025. Reporting followed the Consolidated Criteria for Reporting Qualitative Research (COREQ) [17].

### 2.2. Setting and Participants

The study was conducted at a Grade A tertiary general hospital in Shanghai, China. At the start of data collection, the “One Bed” model had been operating across 11 pilot wards for approximately 48 months. Interviews were conducted in private meeting rooms within the pilot wards. Characteristics of the pilot wards are presented in Table A1 in Appendix A.

Beds in each public-platform ward were centrally managed and allocated by the head nurse. Information on bed availability was accessible through the hospital information system. The head nurse allocated beds according to the admission needs of different specialty teams and coordinated patient admissions with the responsible physicians. Admission to the public-platform wards followed written ward admission rules. These wards primarily admitted patients scheduled for day surgery or other short-stay surgical procedures, generally with an expected length of stay of no more than three days. Patients requiring ongoing management of chronic conditions were generally admitted to the corresponding specialty wards. After admission to a public-platform ward, the patient’s original specialty team retained responsibility for diagnosis, treatment decisions, medical orders, and specialty assessment, while the ward’s fixed nursing team provided daily nursing care.

Each ward was covered by an on-duty physician. Routine clinical issues were managed through the duty system, although the physician was not necessarily continuously present in the public-platform ward. Issues requiring specialist input were communicated to the responsible attending physician through work-group messaging or telephone, whereas urgent concerns were communicated directly by telephone. If the initially contacted physician did not respond promptly, nursing staff sequentially contacted the relevant specialty’s first-line on-call physician, second-line on-call physician, and senior physician or department director. Clinical deterioration was managed immediately according to the hospital’s written emergency-response procedures. When necessary, additional coordination could be sought from the Medical Affairs Department, Nursing Department, or the hospital’s administrative duty office. Written institutional policies covered admission to the public-platform wards, emergency management, and physician duty and on-call arrangements.

Participants were recruited from each of the 11 pilot wards to capture variation in professional role, original specialty, seniority, and implementation experience. Nurses constituted the main participant group because of their continuous involvement in patient reception, daily care, bed coordination, cross-departmental communication, and resource coordination. Physicians were included to provide a complementary medical perspective; the study was not designed for formal comparisons between professional groups. Nurses were eligible if they had worked on the shared-care platform for at least 1 year and held a junior or higher professional title. Physicians were required to have at least 5 years of specialty experience and hold the title of attending physician or above. Head nurses were eligible if they had at least 1 year of management experience and held the title of nurse-in-charge or above. All participants were required to communicate clearly, understand the study, and provide informed consent. Visiting staff and those who had been on leave for more than 6 months were excluded.

LY screened eligible staff and contacted them by telephone. All 25 individuals approached agreed to participate; none declined or subsequently withdrew. Recruitment continued until no substantively new themes or information emerged. The final sample comprised 14 nurses, eight head nurses, and three physicians.

### 2.3. Research Team and Reflexivity

All interviews were conducted by LY (MSc, nurse-in-charge, Department of Nursing, female), who had received formal qualitative research training and had relevant research experience. She had established professional rapport with some participants before the study. However, she was not involved in their clinical care or management and had no role in their supervision, performance appraisal, or treatment decisions. She also had no direct financial relationship with them.

Potential effects of insider status, organizational hierarchy, and social desirability were addressed by conducting interviews privately, emphasizing voluntary participation and confidentiality, and reminding participants that they could decline to answer any question or withdraw without consequence. The interviewer used open-ended questions, clarification, probing, and paraphrasing to elicit participants’ own accounts. She maintained reflexive memos throughout data collection and analysis to examine how professional familiarity, prior assumptions, and organizational hierarchy might influence questioning and interpretation. Independent coding by a second researcher and consensus discussions grounded in the original transcripts provided additional checks on interpretation. These measures were intended to reduce, but could not completely eliminate, possible social-desirability effects.

### 2.4. Interview Guide Development

The research team developed a semi-structured interview guide based on the study objectives, a literature review, and the six RMIC domains. The guide was pilot-tested with one physician, one nurse, and one head nurse to assess clarity, sequence, and relevance. Only minor revisions to wording, question order, and probing prompts were required; the substantive content remained unchanged. Because all three pilot participants met the eligibility criteria, and their interviews addressed the research questions, their data were included in the final analysis. The final interview guide is presented in Table 1.

### 2.5. Data Collection

Individual face-to-face interviews were conducted in a quiet, private setting without other people present. Participants were reminded that participation was voluntary, and that they could decline to answer any question or withdraw at any time. Interviews followed the semi-structured guide, with question order adapted to the interview flow. Clarification, probing, and paraphrasing were used to elicit detailed accounts and confirm meaning.

All interviews were audio-recorded. Field notes documented nonverbal information, including facial expressions, gestures, tone, pauses, and emotional responses. Interviews lasted 30–60 min, with a mean duration of approximately 45 min. Each participant completed one interview; no repeat interviews were conducted. Data collection and preliminary analysis proceeded concurrently. Saturation was assessed across the dataset as a whole and was considered reached through team discussion when subsequent interviews yielded no substantively new themes or relevant information. Saturation was not assessed separately within professional groups.

### 2.6. Data Management and Analysis

LY transcribed each recording verbatim in Chinese within 24–48 h and integrated relevant non-verbal observations from the field notes. A second researcher, HY (MSc, Department of Nursing, female), checked each transcript against the recording. Participants reviewed and were able to correct their transcripts before analysis.

All transcripts were de-identified by removing names, departments, and other identifying information. To reduce the risk of deductive disclosure in this single-center setting, potentially identifying combinations of demographic and professional characteristics were reported only in aggregate. Quotations were labeled by professional role: L for head nurse, N for nurse, and D for physician. Audio recordings, transcripts, and coding files were stored on encrypted devices accessible only to the research team. Data will be retained for 5 years and then securely destroyed.

Data were analyzed in Chinese using the original Chinese transcripts through a sequential hybrid inductive–deductive strategy. In the first stage, inductive qualitative content analysis was conducted to develop codes, categories, subthemes, and themes from participants’ accounts [18,19]. Although the RMIC informed the interview guide and subsequent interpretation, it was not used as a fixed coding template during the initial coding stage. LY and HY independently read the transcripts several times to become familiar with the data and then coded meaningful text segments. Initial codes were compared, discussed, and progressively grouped into categories, subthemes, and themes. After the empirical themes had been developed, the RMIC was used to interpret how the themes and subthemes related to relevant dimensions of integration, including clinical, professional, organizational, system, functional, and normative integration. Themes and subthemes were not required to correspond exclusively to a single RMIC domain. Findings that did not fit clearly within the framework, or that challenged the developing interpretation, were retained and discussed rather than excluded or forced into a predefined category. This subsequent theory-informed interpretation helped the researchers examine how different integration dimensions interacted and where gaps between bed allocation, clinical responsibility, professional roles, support systems, and shared governance remained. NVivo 11 supported data management and coding. Disagreements were resolved by returning to the original transcripts and discussion; unresolved issues were considered by the wider research team.

Quotations selected for inclusion in the manuscript were translated independently from Chinese into English by HY and JF. Any differences in wording or interpretation were discussed with the corresponding author LY until consensus was reached, to ensure conceptual accuracy and consistency with the original Chinese meaning.

Table 2 provides a worked example tracing the analytical progression from a verbatim quotation to initial codes, category, subtheme, theme, and subsequent RMIC mapping, while indicating the coding orientation at each stage.

### 2.7. Trustworthiness

Credibility was supported through pilot testing, iterative probing, field-note documentation, and participant verification of transcripts. Dependability and confirmability were strengthened through independent coding, consensus discussions, and reflexive memoing. Detailed descriptions of the setting, sample, and implementation context were provided to support assessment of transferability. Participants reviewed their transcripts but were not asked to comment on the preliminary themes or final findings.

### 2.8. Ethical Considerations

This study was reviewed by the Medical Ethics Committee of Renji Hospital, Shanghai Jiao Tong University School of Medicine. Based on the study’s nature as hospital management research, the Committee determined that it did not require formal ethics review and issued an exemption (Exemption No. EX-2025-006-2). Before each interview, all participants received information about the study and provided informed consent. Participation was voluntary; participants could decline to answer any question or withdraw at any time without consequence, and all interview data were anonymized before analysis to protect participants’ confidentiality.

## 3. Results

The 25 participants had a mean age of 35.8 years (SD 7.2; range 23–50) and a mean of 13.8 years of work experience (SD 7.9; range 3–31). The sample comprised 14 clinical nurses, eight head nurses, and three physicians and represented surgical, medical, oncology/radiotherapy, emergency/critical care, and clinical research settings. Aggregated participant characteristics are presented in Table 3.

Five themes were identified from the interviews. Role-based review indicated that the professional groups differed mainly in emphasis rather than expressing substantively opposing views. Nursing participants focused on care competence for unfamiliar specialty conditions, workload, physician accessibility, cross-departmental coordination, and perceived safety risks. The three physicians focused more on patient admission and medical management, including bed accessibility, admission efficiency, specialty fit, medical coverage, and nursing capacity. Given the small physician sample, these observations were used to contextualize the findings rather than to support formal interprofessional comparison. These themes describe how participants perceived hospital-wide centralized bed allocation as improving access to inpatient care while also reshaping ward workload, medical response, nursing competence requirements, support processes, and governance responsibilities. To further clarify the relationship between the empirical themes and the RMIC framework, we mapped the five themes and 15 subthemes onto the six RMIC dimensions. The themes did not correspond to the RMIC dimensions in a simple one-to-one manner. Instead, they reflected interactions and misalignment between different dimensions of integration. The mapping further illustrates how the inductively developed themes and subthemes relate to one or more RMIC dimensions. The themes, subthemes, related RMIC dimensions, and corresponding integration gaps are shown in Table 4.

Theme 1. Perceived shorter waiting time and increased ward workload after centralized bed allocation

1.1. Shared beds were perceived to shorten waiting time.

Participants perceived that centralized bed allocation reduced patients’ dependence on beds within their original specialty. Some patients could first be admitted to an available ward and complete examinations, preoperative preparation, and subsequent treatment earlier. One nurse said that patients could *“come in quickly to get prepared, complete examinations, and have surgery earlier”* (N3, nurse). A doctor also noted that *“the admission cycle is shortened” and that “waiting time can also be reduced”* (D1, doctor). Participants generally viewed centralized bed allocation as improving timely access to inpatient care.

1.2. Faster bed turnover was perceived to make ward work more intensive.

Participants felt that shared beds reduced unused bed capacity and allowed more patients to be admitted. At the same time, the same bed was used for repeated admissions and discharges within a shorter period. Participants described the pace of ward work as becoming faster. Nurses had to conduct admission assessments, preoperative education, documentation, discharge guidance, and bed preparation more frequently. One nurse stated that *“the patient turnover rate is higher” and that “the pace of work is also very fast”* (N1, nurse). A head nurse also noted that *“the one-bed-for-the-whole-hospital model can improve bed utilization”* (L1, head nurse). These findings suggest that faster bed turnover was not perceived only as increasing admission capacity; it also required wards to complete more repeated care processes within a shorter time.

1.3. Single efficiency indicators were perceived as insufficient for reflecting actual workload.

Participants felt that bed occupancy, length of stay, or surgical volume alone could not fully reflect the actual workload of the shared platform. Complex cases often required longer hospitalization and could make turnover indicators appear less favorable. As one doctor stated, *“our patients occupy beds for at least two weeks longer than theirs,” so “the indicators are not ideal”* (D1, doctor). In contrast, short-stay patients occupied beds for less time, but admission assessment, preoperative preparation, education, documentation, and discharge coordination did not decrease proportionally. Some cross-specialty surgeries and nursing tasks were also not fully included in current performance statistics. One nurse noted that *“not all surgeries are included in our scope”* (N2, nurse). As a result, there was a gap between measured efficiency and the workload experienced by the ward.

Overall, centralized bed allocation was perceived to improve access to inpatient care and increase bed use. At the same time, participants perceived it as making ward work more intensive, and the findings suggest that single efficiency indicators may not adequately capture nursing input or case complexity.

Theme 2. Patients were perceived to move across wards faster than medical response followed

2.1. Patient location changed, but responsible doctors were not always perceived as present or accessible.

Patients could be admitted to wards outside their original specialty. However, participants reported that responsible doctors still mainly worked in their own specialty units. When a patient’s condition changed, the receiving ward often had to contact the original specialty team. Participants perceived that doctors in the receiving ward might be unfamiliar with the patient or might have limited authority to issue specialty-related medical orders. A head nurse said that, in some situations, *“none of the three doctors dared to issue medical orders”* (L1, head nurse). A nurse also stated that, in emergencies, *“we could not provide emergency treatment and could only measure vital signs”* (N2, nurse). These accounts reflected nursing staff’s perception that the physical transfer of patients was not always accompanied by equally accessible on-site medical decision-making.

2.2. Delayed medical response was perceived to increase nurses’ cross-specialty communication work.

When responsible doctors were not readily available in the receiving ward, nurses reported contacting them by telephone, work-group messaging, or by going to other floors. Participants attributed difficulties in reaching responsible doctors to circumstances such as surgery, ward rounds, or delayed responses to messages, which sometimes required nurses to contact on-call staff or other departments repeatedly. One head nurse said that *“many urgent issues require phone calls to surgeons” and that nurses “often spend a lot of time communicating”* (L4, head nurse). A nurse also noted that *“doctors are difficult to reach” and that “messages in the group cannot be replied to in time”* (N12, nurse). As a result, nursing work extended beyond direct care. It also included tracking information, relaying problems, and urging decisions.

2.3. Lower doctor visibility was perceived by staff to affect patients’ sense of safety.

Participants reported observing that some patients appeared uncertain or concerned when they could not see their responsible doctor promptly. Participants observed that these reactions became more apparent when patients were unable to see a doctor for an extended period or when their needs had to be relayed repeatedly through nurses. A head nurse described these patients as *“not like someone’s own children, but left here”*, and said that they *“lack a sense of belonging”* (L3, head nurse). A nurse also noted that “when patients come in and cannot see doctors, they lack a sense of safety” (N3, nurse). These findings reflect staff observations and interpretations of patients’ reactions, rather than direct patient accounts.

Overall, participants perceived that patient placement changed more rapidly than medical availability, responsibility pathways, and cross-specialty coordination mechanisms adapted. Nurses therefore took on more cross-specialty communication and coordination, while also responding directly to patients’ concerns and uncertainty. These findings primarily reflect the experiences and observations of nurses and head nurses and should not be interpreted as direct evidence of patients’ experiences or as a comprehensive account of physicians’ own workload, responsibilities, or organizational constraints.

Theme 3. Cross-specialty care demands were perceived to exceed what nurses could manage through temporary learning

3.1 Expanded case mix was perceived to increase gaps in nursing competence and safety pressure.

Participants described shared beds as expanding the range of diseases, treatments, and risks that nurses had to manage. Nurses need to recognize different observation priorities, manage specialty-specific tubes and devices, provide perioperative care, and respond to emergencies. Participants did not attribute this problem simply to individual nurses’ lack of ability. Instead, they emphasized that staffing structure, specialty support, and learning conditions had not changed at the same pace as the patient mix. Junior nurses felt more uncertain when facing unfamiliar changes in patients’ conditions. One nurse said, “*without understanding disease knowledge, I do not feel confident that I can provide adequate care*” (N13, nurse). A head nurse also noted that the team was mainly composed of junior and mid-level nurses, and that “*in terms of nursing safety, it was indeed very difficult at the beginning*” (L1, head nurse).

3.2. Insufficient preparation made learning while working the main way nurses adapted.

When formal preparation was insufficient, learning was embedded in daily admission work. Nurses searched for information, consulted the original specialty, or invited staff from relevant departments to give teaching according to the patients they had already received. One nurse said, “We *learn the specialty knowledge of the corresponding department based on which patients are admitted to the shared platform*” (N4, nurse). A head nurse also stated that there was no systematic preparation at first, and that they “*asked relevant departments during the process of exploration and invited them to teach related courses*” (L3, head nurse). This problem-driven learning appeared to help teams deal with immediate tasks. However, it depended on cases that had already appeared and they could not build stable and complete cross-specialty competence before admission.

3.3. Tiered mentoring, core groups, and shared specialty support were viewed as important organizational support.

Participants felt that one-off slide-based training could not fully cover real care situations. Simulation, on-site support from senior nurses, and core group collaboration were viewed as more useful for translating specialty knowledge into clinical judgment. One nurse said that “*simulation should be more effective than simple lectures and PowerPoint presentations*” (N1, nurse). Another nurse suggested that a “*core group can manage together through collaboration*” (N9, nurse). For problems that occurred less often but required high specialty expertise, some head nurses suggested sharing existing specialist nurse resources across the hospital, rather than requiring each shared platform to develop all specialty capabilities independently. These findings describe the types of support participants expected. They do not show that these approaches were effective in this study.

Overall, cross-specialty admissions changed the competence required of nurses. Temporary learning appeared to help nurses respond to some problems, but participants described this approach as unstable. These findings suggest that cross-specialty care cannot rely only on individual adaptation. It may require tiered training, on-site support, and accessible specialty resources.

Theme 4. Support systems were perceived as lagging behind patient movement

4.1. Information and logistical processes were perceived as not moving with patients.

After the shared platform was introduced, some information system permissions, examination processes, and logistical services were not connected in time. Patients had already moved to receiving wards, but medication, linen, examinations, and delivery processes still followed previous departmental boundaries. A head nurse said that “*the newly opened system could not match logistics and the radiology department*” (L1, head nurse). A nurse also noted that some medications “*would not arrive until 2:00 p.m.*” (N2, nurse), which was described as disrupting the day’s nursing arrangements. Beds could be centrally allocated, but the information and logistical processes supporting these beds remained fragmented. Nurses therefore had to contact related departments repeatedly to keep examinations, medication use, and daily care moving.

4.2. Space, equipment, and supplies were perceived as not always matching cross-specialty needs.

Some receiving wards were adapted from existing spaces. Transfer routes, bedside observation conditions, and basic facilities were not always designed for inpatient care. Patients from different specialties also required different functional beds, special instruments, and dedicated supplies. Allocating patients based only on bed availability could therefore exceed the ward’s existing capacity. One nurse said that “it is difficult for surgical transfer beds to move in and out” and that “*the safety problem is very serious*” (N1, nurse). Another nurse noted that “*some orthopedic instruments are too specialized*” (N9, nurse). These accounts show that bed availability was not only a matter of whether a bed was empty. It also depended on whether the space, equipment, and supplies could support the care required by that specialty.

4.3. Nurses described manual coordination as necessary but poorly visible work.

To distinguish patients from different specialties and care pathways, nurses had to add manual labels to examination forms, handover information, and bedside materials. They also had to repeatedly confirm examination locations, medical orders, and sources of supplies. One nurse said, *“We need to distinguish that this is a cardiology patient” and mark it “on every examination form”* (N4, nurse). This work was described as helping to maintain continuity in cross-specialty care, but it was difficult to capture through routine nursing items or performance indicators. This was especially clear in short-stay and high-turnover settings. Shorter bed occupancy did not mean fewer nursing processes. As one head nurse stated, *“even if the patient stays for only half a day”, the nursing workflow is almost the same as for “a full day”* (L8, head nurse). Thus, part of the coordination work was absorbed into daily nursing practice but was not fully recognized as actual workload.

Overall, centralized bed allocation changed how patients moved across wards. However, information systems, logistics, space, equipment, and supplies did not always change at the same pace. Participants described nurses as maintaining workflow through phone calls, manual labeling, borrowing supplies across departments, and repeated checking. This work was presented as important for continuity of care, but it often remained invisible.

Theme 5. Unclear boundaries and governance responsibilities were perceived to limit sustained bed sharing

5.1. The scope of the model, specialty grouping, and admission criteria were perceived as needing clearer boundaries.

Participants did not believe that all empty beds could receive all patients without distinction. They first questioned what the “one-bed-for-the-whole-hospital” model should cover and how deeply specialties should share beds. Participants worried that completely removing all specialty boundaries could lead to an overly mixed case mix. They were also worried that this could exceed the receiving ward’s staffing capacity, equipment conditions, and ability to manage risk. One head nurse said that *“the coverage of the one-bed-for-the-whole-hospital model must be clarified at the beginning”* (L5, head nurse). Another head nurse also noted that *“each shared platform needs to know which diseases it admits”*, because the case mix “cannot be too chaotic” (L8, head nurse). Therefore, grouping related specialties, defining eligible conditions, and excluding critically ill patients were viewed as important ways to use beds more flexibly while keeping care risks within a manageable range.

5.2. Bed allocation was perceived as needing to consider severity, specialty fit, and nursing workload.

Participants felt that the number of empty beds alone could not determine whether a bed was suitable for a patient. Disease severity, surgical risk, observation intensity, specialty equipment needs, and the nursing workload on that shift were all perceived to affect whether placement was safe. One nurse said that, *for “relatively major surgeries,” patients might need to be placed “a little closer to the nurses”* (N9, nurse). Another nurse noted that *the shared platform “basically admits patients undergoing grade IV surgery”* (N10, nurse). These accounts suggest that centralized allocation should not only identify where beds are available. It also needs to assess whether the receiving ward can carry the related risks and nursing tasks at that time.

5.3. Responsible leadership, mobilizable resources, and incentives were perceived as needing alignment.

A shared platform could serve several specialties at the same time, but its fixed management authority was not always clear. Head nurses often handled daily coordination, but they did not necessarily have the authority to mobilize doctors, equipment, budgets, or assets. One head nurse said that *“the shared platform does not have a department director,” so the team’s “sense of belonging is weaker”* (L8, head nurse). Another head nurse asked *whether staff could receive “some income support” when workload could not be reduced* (L1, head nurse). These findings suggest that if workload, risk, and performance support are not aligned, staff may feel that the arrangement is unfair or difficult to sustain. A few participants also mentioned unclear nurse entry requirements, risk compensation, and career pathways. These views further point to the need for clearer governance arrangements.

Overall, bed sharing was not simply a matter of pooling empty beds. Participants described a need for clear decisions about which patients were suitable for shared platforms, which wards could manage which conditions, and who had the authority to coordinate medical, nursing, equipment, and performance resources. The present data cannot prove that these conditions determine long-term sustainability. However, they show that staff viewed them as important prerequisites for reducing risk and maintaining collaboration.

## 4. Discussion

Hospital-wide bed sharing should be understood as an uneven process of integration, not merely as a bed-allocation strategy. Participants perceived that centralized allocation improved access to beds, but clinical, professional, functional, organizational, and normative integration did not keep pace. Patients moved across wards, while medical response, nursing readiness, functional support, workload recognition, and governance remained partly organized around specialty boundaries. Previous qualitative studies of bed sharing and the placement of patients outside specialty-appropriate wards have reported similar concerns. These include heavier nursing workload, communication barriers, changes in physicians’ work, clinical risks, and disruption of specialty-based nursing practice [6,20]. The present study extends this work by linking these concerns to a common mismatch between bed integration and care integration. It also identifies nurses’ manual and often invisible coordination work as a mechanism that helped fragmented work systems continue to function. The study therefore provides an explanatory account of how perceived operational flexibility may coexist with incomplete care integration. It does not establish effects on operational or patient outcomes.

### 4.1. Work Redistribution and the Limits of Efficiency-Based Evaluation

This uneven integration first appeared in the redistribution of work. Bed allocation may improve patient flow and resource use, but it does not remove work; it redistributes work across settings [21]. Participants’ accounts suggested that earlier admission was accompanied by a transfer of assessment, monitoring, and coordination tasks to receiving wards. If evaluation focuses only on bed occupancy, length of stay, turnover, waiting time, or surgical volume, efficiency gains may be overstated, while nursing workload, case complexity, and coordination costs may be underestimated [22]. Across diverse healthcare systems, qualitative evidence consistently links staff shortages, heavy clinical workloads, insufficient organizational support, and blame for system failures with unfinished care, psychological distress, and professional dissatisfaction. These convergent findings suggest that systemic resource constraints can generate similar perceptions of clinical risk among frontline staff, even when the organizational contexts differ [23,24]. Viewed through the RMIC, this pattern suggests that system integration in bed allocation outpaced functional integration in workload recognition and staffing support. Implementation should therefore align workload recognition and staffing support with explicit escalation pathways [13,25].

### 4.2. Patient Relocation, Clinical Responsibility, and Medical Response

Participants described spatial relocation without corresponding integration of clinical responsibility: patients moved to another ward, while responsible physicians often remained embedded in their original specialty teams, routines, and duty systems. Studies of bed-spaced patients and atypical intrahospital transfers have reported differences in care processes, specialist review, length of stay, and downstream outcomes, although mortality findings are inconsistent [26,27,28]. In RMIC terms, organizational integration of bed allocation appeared to outpace clinical and professional integration. Receiving nurses consequently became intermediaries who sought clarification, contacted specialty physicians, and managed uncertainty while awaiting decisions. International qualitative evidence similarly indicates that nurses may be left responsible for deteriorating patients when ward physicians are difficult to reach. Shared clinical language, rapid-response support, and cross-ward competence transfer may partially bridge this gap, although their effectiveness remains constrained by time and resources [29]. Bed sharing should therefore include clear arrangements for physician availability, decision authority, duty coverage, specialty consultation, and escalation when patient risks emerge.

Unresolved clinical responsibility also has professional and medico-legal implications. When accountability is ambiguous, frontline staff may feel exposed to complaints or legal consequences for failures that they cannot fully control. Across healthcare settings, poorly supported accountability processes have been associated with anxiety, loss of professional confidence, and defensive practice [30]. Nationwide epidemiological evidence from Kazakhstan further underscores the need for state-level monitoring of medical errors and their consequences, providing a system-level comparative perspective on clinical and legal risks within healthcare systems [31]. Although these data do not establish a direct association between cross-ward admission and adverse events, they provide a relevant system-level comparison. Rapid operational change should therefore be accompanied by clear institutional responsibility, structured incident review, and aggregated safety surveillance. Such arrangements may protect patients while reducing the unfair individualisation of failures arising from system design.

### 4.3. Cross-Specialty Nursing Demands and Professional Support

Participants framed cross-specialty care as a gap between expanding care demands and organized professional support, rather than simply as insufficient nursing competence. Evidence from floating nursing likewise suggests that work in unfamiliar clinical areas can increase uncertainty, disrupt routines, and raise safety concerns [32,33]. Qualitative evidence concerning “outlier patients,” who are cared for outside specialty-appropriate wards, similarly suggests that such placement can disrupt specialty-based nursing practice and undermine nurses’ confidence and professional morale [20]. Participants described maintaining care through workplace learning, specialty consultation, and information seeking. Although this reflects professional adaptability, reliance on informal adaptation is difficult to sustain. In RMIC terms, cross-specialty demands expanded faster than professional and organizational integration. Structured competencies and accessible specialty expertise are therefore needed [13,14].

### 4.4. Functional Support Systems and Invisible Coordination Work

Bed-level integration also outpaced functional integration. Although beds were shared, information, medication delivery, examination scheduling, equipment, supplies, and logistical support did not always follow the patient. Nurses compensated through manual coordination, including reconciling information across systems, tracking delayed services, and sourcing equipment. These accounts position nurses as boundary spanners across wards, specialties, and support services [34,35], consistent with evidence that much nursing coordination remains invisible in routine workload measures [36,37,38]. In RMIC terms, this manual coordination was not merely an additional task; it was a mechanism that sustained fragmented work systems when functional integration remained incomplete. Hospital-wide bed sharing therefore requires functional infrastructure and workload measures that recognize coordination work [13].

### 4.5. Bounded Flexibility, Governance, and Conditions for Sustained Bed Sharing

Bed sharing should be understood as bounded flexibility rather than unrestricted openness. Participants generally did not reject hospital-wide bed allocation itself; instead, they emphasized that an empty bed is not necessarily a suitable bed [39]. Participants perceived that bed sharing could improve flexibility and reduce admission waiting; however, these operational effects were not directly measured in this study. They also perceived that appropriate cross-ward placement depended on whether the receiving ward had the required specialty knowledge, equipment, staffing, and capacity to manage patient risk. When patients with high acuity, complex postoperative needs, special devices, or intensive specialty care are placed in wards without adequate support, bed sharing may shift clinical risk and coordination burden to frontline staff. In this context, boundaries appear less as barriers to bed sharing than as conditions for sustaining it [6]. These boundaries reflect both organizational and normative integration. Organizational integration is needed to define responsibility, resource authority, escalation pathways, and workload-sensitive staffing support. Normative integration is needed to establish shared admission criteria, exclusion rules, accountability, and cross-departmental consensus [11]. Thus, hospital-wide bed sharing should not be implemented by simply opening all beds to all patients. It should be governed through explicit patient selection, clear responsibility structures, and resource mechanisms that match bed availability with clinical suitability.

### 4.6. From Capacity Pooling to Safe Clinical Capacity: An International Synthesis

Taken together, our findings connect hospital-wide bed sharing to the broader literature on organizational flexibility, capacity pooling, and patient flow. In healthcare, operational flexibility has been conceptualized as a multidimensional capability involving physical, human-resource, volume, behavioral, and conceptual dimensions, through which organizations adapt to variable demand [40]. Patient-flow research further conceptualizes hospitals as interdependent service systems and shows that routing and transfer arrangements are closely related to delays, workload distribution, and fairness [41]. Hospital-wide bed sharing can therefore be viewed as a form of flexible capacity allocation, through which beds designated for one specialty may be used by patients from another [42,43]. However, hospital-specific evidence suggests that the benefits of capacity pooling are conditional. Off-service placement may make otherwise unavailable capacity accessible, but it has also been associated with a longer remaining length of stay and a higher likelihood of 30-day readmission [42]. Modeling research on flexible bed allocation similarly suggests that full flexibility can be difficult to manage, may reduce the benefits of specialization, and may require extensively multi-skilled clinical teams. Appropriately designed partial flexibility may retain much of the benefit associated with economies of scale [43]. Clustered overflow arrangements have accordingly been proposed to combine dedicated and pooled capacity while limiting the adverse effects of unrestricted pooling and the costs of cross-specialty training [44]. Our findings extend this literature by explaining why operational flexibility may not automatically translate into safe and usable clinical capacity. Participants did not perceive a hospital bed as a homogeneous unit of capacity. Its effective use also required timely medical response, appropriate nursing competence, information and logistical support, suitable equipment, and clear authority and accountability. When physical beds were pooled without corresponding integration of these clinical and organizational resources, additional coordination work, workload, and clinical risk were perceived to shift to receiving wards. Bounded flexibility therefore connects the operations management logic of capacity pooling with the RMIC perspective. Capacity pooling explains why hospitals share beds, whereas integrated care identifies the clinical, professional, functional, organizational, and normative conditions required to convert pooled beds into safe and usable clinical capacity.

### 4.7. Implications for Practice

These findings suggest that hospital-wide bed sharing should be implemented as a care model, not only as a bed-allocation strategy. First, evaluation should extend beyond system-level indicators such as bed occupancy, turnover, waiting time, and surgical volume. Nursing workload, case complexity, coordination work, professional well-being, and patient safety risks should also be measured. Second, cross-ward admission should be guided by clinical suitability rather than bed vacancy alone. Before placement, hospitals should consider disease severity, specialty fit, staffing, equipment, observation needs, and the ward’s capacity to respond to deterioration. Third, clinical and professional responsibilities should be explicit. Every cross-ward patient should have a named responsible medical team, documented decision authority, defined duty coverage, accessible consultation routes, and measurable escalation and response standards. Fourth, nurses in receiving wards need structured professional support rather than relying mainly on informal learning. Stratified training, simulation, specialist nurse consultation, core support groups, standardized emergency procedures, and accessible expert resources may therefore be needed. Finally, cross-ward adverse events, near misses, delayed medical responses, and failures of clinical ownership should be incorporated into non-punitive institutional monitoring. Aggregated indicators should inform regional or state-level surveillance where appropriate, supporting organizational learning while protecting both patients and healthcare workers from system-generated risks.

### 4.8. Limitations

This study has several limitations. First, this was a single-center study conducted in a well-resourced Grade A tertiary hospital in Shanghai. The hospital’s staffing capacity, digital infrastructure, specialist availability, bed-management system, and organizational support may not reflect conditions in lower-tier hospitals or less-developed regions. However, resource constraints alone do not necessarily mean that the challenges of centralized bed allocation will be more severe. In some lower-tier hospitals, lower inpatient demand or bed occupancy may reduce the practical need for cross-specialty bed pooling; in other settings, high demand combined with limited staffing, weaker information systems, or reduced specialist support may make implementation more difficult. Implementation was also shaped by the organizational and policy context of this Chinese tertiary hospital, including specialty-based clinical responsibility, centralized administrative coordination, and hospital-specific arrangements for nursing and medical management. Transferability therefore depends on the interaction among bed-demand pressure, resource capacity, hospital level, and local organizational arrangements and should be examined across hospitals in different regions and socioeconomic contexts. Second, the study mainly captured the perspectives of healthcare professionals involved in hospital-wide bed sharing. Patients and family members were not interviewed. Their experiences of cross-ward admission, perceived safety, communication, and continuity of care were therefore not directly examined. Accordingly, findings concerning patients’ sense of safety, sense of belonging, and reactions to cross-ward admission should be interpreted as staff observations and interpretations rather than direct patient accounts. This also limits the study’s ability to assess person-centered, clinical, and normative integration from the patient perspective. Third, purposive recruitment may have introduced selection bias. Although participants were recruited from all 11 pilot wards to capture variation in professional role, specialty, seniority, and implementation experience, the sample may not represent eligible staff who were not approached or who may have held different views of the model. Physicians were also underrepresented, with only three physicians interviewed, whereas most participants were nurses or head nurses. Information sufficiency was assessed across the dataset as a whole rather than within individual professional groups; subgroup-specific saturation was therefore not established. The findings therefore provide a more detailed account of nursing staff’s experiences of cross-ward coordination than of physicians’ firsthand experiences. In particular, the study could not fully examine physicians’ workload, duty arrangements, clinical decision-making responsibilities, or organizational constraints. Findings concerning medical availability and response should therefore be interpreted primarily as participants’ perceptions and observations. Future studies should recruit a broader range of physicians and compare experiences across professional groups. Fourth, the findings were based on interview data. The study did not directly measure waiting time, bed use, workload, safety events, patient outcomes, or patient experiences. Future studies should combine staff interviews with operational indicators, workload measures, and direct accounts from patients and family members. Finally, the study focused on implementation challenges and practice experiences at one stage of bed sharing. Bed-sharing models may change over time as information systems, staffing arrangements, and cross-departmental rules improve. Longitudinal research is needed to examine how these models evolve and whether the proposed support mechanisms improve safety and care coordination.

## 5. Conclusions

This study suggests that hospital-wide centralized bed allocation should be understood as an uneven process of integration rather than only as a bed-management strategy. These findings primarily reflect the perceptions and experiences of nurses and nursing managers, supplemented by limited physician input. Across five themes, participants described a consistent mismatch: beds could be pooled and patients could be moved rapidly, but medical response, nursing competence, information and logistical support, workload recognition, and governance arrangements did not always move at the same pace. This gap helps explain why improved access to beds may coexist with heavier nursing workloads, delayed cross-specialty coordination, and uncertainty about clinical responsibility. The findings extend previous qualitative work by showing that the central challenge is not bed sharing itself, but the misalignment between bed integration and care integration. Sustainable implementation therefore requires bounded flexibility: bed allocation should be guided not only by vacancy, but also by clinical suitability, specialty fit, nursing workload, timely medical response, functional support, and shared accountability.

## Figures and Tables

**Table 1:** Interview Guide.

Level	Dimension	Interview Questions
Macro level	System integration	How do you understand the “one-bed-for-the-whole-hospital” model? In your view, why is the hospital promoting this model?
		What effects do you think this model may have on admission waiting time, bed use, and hospital operations?
		In terms of policy, medical insurance, information systems, or external resources, what factors may support or limit the implementation of this model?
Meso level	Organizational integration	What preparations did your department make before implementing or piloting the “one-bed-for-the-whole-hospital” model?
		During cross-ward admission, how are responsibilities divided between departments? Which responsibilities are clear, and which remain unclear?
		What support do you think the hospital still needs in terms of organizational structure, management systems, or collaboration mechanisms?
	Professional integration	Under this model, what roles are played by doctors, nurses, managers, the information technology department, logistics staff, and other professionals?
		After cross-specialty patients are admitted, how do doctors and nurses communicate and collaborate? Which steps are most difficult?
		When the receiving ward encounters unfamiliar specialty-related problems, how does it usually obtain specialty support?
Micro level	Clinical integration	Please recall one experience of admitting or caring for a cross-ward patient. What was the main care process from admission to discharge?
		During cross-ward care, how are patient assessments, clinical observations, treatment support, examination scheduling, and discharge preparation completed?
		From your observation, what reactions, questions, or concerns do patients or family members have about cross-ward admission?
		What impact do you think this model has on patient safety, continuity of care, and patient-centered nursing?
Supportive integration	Functional integration	In actual practice, do information systems, bed scheduling, medication delivery, examination scheduling, supplies, equipment, and logistical support meet the needs of cross-ward care?
		Which support systems work smoothly? Which steps require additional coordination by nurses or departments?
		Does this additional coordination affect nursing workload, work efficiency, or patient care?
	Normative integration	In your view, what admission criteria or exclusion criteria should guide cross-ward admission? Which patients are suitable or unsuitable for cross-ward admission?
		When a patient’s condition changes, medical orders are unclear, or responsibility is ambiguous, how is the situation usually handled?
		Which systems, rules, or responsibility boundaries still need to be clarified?
Summary	Suggestions for improvement	Overall, what do you think are the main advantages of the “one-bed-for-the-whole-hospital” model? What are the main difficulties?
		If this model is further promoted in the future, what should be improved first?
		Is there any other experience or suggestion you would like to add regarding this model?

**Table 2:** Worked example of the analytic process and RMIC mapping.

Analytical Step	Worked Example	Coding Orientation
Verbatim quotation	“For many urgent matters, we have to call the surgical doctors… nurses spend a great deal of time communicating.” (L4)	Source data
Initial codes	No on-site physician; delayed response; unclear responsibility; communication burden; emergency risk	Inductive
Category	Disconnection between bed integration and medical responsibility	Inductive
Subtheme	Nurses became communication intermediaries	Inductive
Theme	Theme 2: Medical responsibility lagged behind patient movement, disrupting continuity	Inductive
RMIC mapping	Clinical integration; professional integration; organizational integration	Deductive, theory-informed interpretation after theme development

Note: The progression from the quotation to the initial codes, category, subtheme, and theme was inductive. RMIC was applied only after theme development as a deductive, theory-informed interpretive framework. The theme was allowed to map to more than one RMIC dimension.

**Table 3:** Aggregated characteristics of participants (*n* = 25).

Characteristic	Category/Statistic	n (%) or Value
Sex	Female	23 (92.0)
	Male	2 (8.0)
Age, years	Mean (SD); range	35.8 (7.2); 23–50
Education	Junior college	7 (28.0)
	Bachelor’s degree	14 (56.0)
	Master’s degree	1 (4.0)
	Doctoral degree	3 (12.0)
Professional role	Clinical nurse	14 (56.0)
	Head nurse	8 (32.0)
	Physician	3 (12.0)
Professional title group	Registered/senior nurse	12 (48.0)
	Nurse practitioner/ nurse-in-charge/ intermediate title	10 (40.0)
	Attending/associate chief physician	3 (12.0)
Work experience, years	Mean (SD); range	13.8 (7.9); 3–31
Specialty group	Surgical specialties	13 (52.0)
	Medical specialties	8 (32.0)
	Oncology or radiotherapy	2 (8.0)
	Other	2 (8.0)

**Table 4:** Themes, subthemes, and RMIC dimensions.

Theme/Subtheme	Related RMIC Dimension(s)	Integration Gap Reflected
Theme 1. Centralized bed allocation was perceived to shorten waiting time but increase ward workload	System integration; functional integration	Bed resources were centrally pooled to improve access and patient flow, but workload was redistributed to receiving wards.
1.1. Shared beds were perceived to shorten waiting time	System integration	Bed resources were pooled beyond ward ownership to improve access and patient flow.
1.2. Faster bed turnover was perceived to make ward work more intensive	System integration; functional integration	Faster patient movement increased bedside workload and coordination demands.
1.3. Single efficiency indicators were perceived as insufficient for reflecting actual workload	Functional integration; normative integration	Bed-use indicators did not capture nursing workload, case complexity, or hidden coordination work.
Theme 2. Patients were perceived to move across wards faster than medical response followed	Clinical integration; professional integration; organizational integration	Patient placement changed faster than clinical responsibility, medical response, and professional coordination mechanisms.
2.1. Patient location changed, but responsible doctors were not always perceived as present or accessible	Clinical integration; organizational integration	Patient placement changed faster than responsibility and medical response pathways.
2.2. Delayed medical response was perceived to increase nurses’ cross-specialty communication work	Professional integration; clinical integration	Nurses became intermediaries between patients, receiving wards, and original specialty teams.
2.3. Lower doctor visibility was perceived by staff to affect patients’ sense of safety	Clinical integration; normative integration	Reduced visible medical presence weakened perceived continuity and trust in cross-ward care.
Theme 3. Cross-specialty care demands were perceived to exceed what nurses could manage through temporary learning	Professional integration; organizational integration	Expanded case mix required structured competence-building beyond ad hoc individual adaptation.
3.1. Expanded case mix was perceived to increase gaps in nursing competence and safety pressure	Professional integration; clinical integration	Nurses were expected to care for patients beyond their usual specialty scope, creating perceived safety pressure.
3.2. Insufficient preparation made learning while working the main way nurses adapted	Professional integration; organizational integration	Cross-specialty competence depended on individual learning rather than structured preparation.
3.3. Tiered mentoring, core groups, and shared specialty support were viewed as important organizational support	Professional integration; organizational integration	Participants emphasized the need for structured capability-building and accessible specialist support.
Theme 4. Support systems were perceived as lagging behind patient movement	Functional integration; organizational integration; professional integration	Information, logistics, space, and materials remained fragmented and were compensated for by nursing coordination.
4.1. Information and logistical processes were perceived as not moving with patients	Functional integration	Information access, testing processes, medication delivery, and logistical support remained fragmented.
4.2. Space, equipment, and supplies were perceived as not always matching cross-specialty needs	Functional integration; organizational integration	Receiving wards did not always have the material conditions required for cross-specialty care.
4.3. Nurses described manual coordination as necessary but poorly visible work	Functional integration; professional integration; normative integration	Nurses absorbed system fragmentation through boundary-spanning coordination that was weakly recognized.
Theme 5. Unclear implementation boundaries and governance responsibilities were perceived to limit sustained bed sharing	Normative integration; organizational integration	Bed sharing requires clearer shared rules, authority, resources, and accountability.
5.1. The scope of the model, specialty grouping, and admission criteria were perceived as needing clearer boundaries	Normative integration; organizational integration	Bed sharing lacked explicit rules about eligible patients, specialty boundaries, and admission conditions.
5.2. Bed allocation was perceived as needing to consider severity, specialty fit, and nursing workload	Clinical integration; functional integration; normative integration	An empty bed was not necessarily a suitable bed without matching clinical risk, specialty needs, and nursing capacity.
5.3. Responsible leadership, mobilizable resources, and incentives were perceived as needing alignment	Organizational integration; normative integration	Sustainable bed sharing requires clear governance, resource authority, and shared accountability.

Note. Themes and subthemes were developed inductively from participants’ accounts and then mapped onto relevant RMIC dimensions. The final column summarizes the integration gap reflected by each theme or subtheme.

## Data Availability

The datasets generated and analyzed during the current study are not publicly available due to privacy and ethical restrictions related to the qualitative interview data. De-identified data may be available from the corresponding author upon reasonable request and with permission from the relevant ethics committee.

## Abbreviations

The following abbreviations are used in this manuscript:

COREQ	Consolidated Criteria for Reporting Qualitative Research
RMIC	Rainbow Model of Integrated Care
WHO	World Health Organization

## Appendix A

**Table A1:** Characteristics of public-platform wards involved in hospital-wide bed sharing.

Domain	Characteristic	Revised Value
Nursing workforce	Nurses on the formal staffing roster	153
	On-duty nurses/active workforce	151
	Nurses in standardized training	28/151 (18.5%)
Educational level	Master’s degree or above	3/151 (2.0%)
	Bachelor’s degree	89/151 (58.9%)
	Junior college diploma	58/151 (38.4%)
	Secondary vocational diploma	1/151 (0.7%)
Professional title	Registered nurse	65/151 (43.0%)
	Senior nurse	71/151 (47.0%)
	Nurse-in-charge	15/151 (9.9%)
	Associate chief nurse or above	0/151 (0.0%)
Work experience	≤1 year	9/151 (6.0%)
	>1–5 years	46/151 (30.5%)
	>5–10 years	43/151 (28.5%)
	>10–20 years	42/151 (27.8%)
	>20 years	11/151 (7.3%)

Note. Data were derived from an internal Nursing Department survey of the 11 public-platform wards. Ward names, campus names, and nurse manager names were removed for anonymity. “Approved beds” refers to the formally registered bed complement assigned to these wards (n = 326). “Open beds” refers to beds actually in service at the time of the survey (n = 367), including 41 temporary or additional beds opened in response to clinical demand. “Nurses on the formal staffing roster” represents the administrative workforce count (n = 153), whereas “on-duty nurses” represents the active workforce in the pilot wards at the time of the survey (n = 151). Nurses participating in standardized training were included in the active workforce. Educational level, professional title, and work-experience categories use on-duty nurses as the denominator (n = 151).
